# *Trichinella spiralis* Infection Inhibits the Efficacy of RBD Protein of SARS-CoV-2 Vaccination via Regulating Humoral and Cellular Immunity

**DOI:** 10.3390/vaccines12070729

**Published:** 2024-06-30

**Authors:** Feifan Zhu, Wenwen Zheng, Yiyan Gong, Jinyuan Zhang, Yihan Yu, Jixian Zhang, Mengjun Liu, Fei Guan, Jiahui Lei

**Affiliations:** 1 Department of Pathogen Biology, School of Basic Medicine, Tongji Medical College and State Key Laboratory for Diagnosis and Treatment of Severe Zoonotic Infectious Diseases, Huazhong University of Science and Technology, Wuhan 430030, China; d202381758@hust.edu.cn (F.Z.); m202275408@hust.edu.cn (W.Z.); m202375568@hust.edu.cn (Y.G.); zhangjinyuan@hust.edu.cn (J.Z.); 2 Department of Pulmonary Medicine, Hubei Provincial Hospital of Integrated Traditional Chinese and Western Medicine, Wuhan 430015, China; yuyihan2000@126.com (Y.Y.); jxzhang1607@163.com (J.Z.); 18808665690@163.com (M.L.)

**Keywords:** *T. spiralis*, COVID-19, vaccine efficacy, GC B cells, Th2, Treg, ALB

## Abstract

Vaccines are the most effective and feasible way to control pathogen infection. Helminths have been reported to jeopardize the protective immunity mounted by several vaccines. However, there are no experimental data about the effect of helminth infection on the effectiveness of COVID-19 vaccines. Here, a mouse model of trichinosis, a common zoonotic disease worldwide, was used to investigate effects of *Trichinella spiralis* infection on the RBD protein vaccine of SARS-CoV-2 and the related immunological mechanism, as well as the impact of albendazole (ALB) deworming on the inhibitory effect of the parasite on the vaccination. The results indicated that both the enteric and muscular stages of *T. spiralis* infection inhibited the vaccine efficacy, evidenced by decreased levels of IgG, IgM, sIgA, and reduced serum neutralizing antibodies, along with suppressed splenic germinal center (GC) B cells in the vaccinated mice. Pre-exposure to trichinosis promoted Th2 and/or Treg immune responses in the immunized mice. Furthermore, ALB treatment could partially reverse the inhibitory effect of *T. spiralis* infection on the efficiency of the vaccination, accompanied by a restored proportion of splenic GC B cells. Therefore, given the widespread prevalence of helminth infections worldwide, deworming therapy needs to be considered when implementing COVID-19 vaccination strategies.

## 1. Introduction

The Corona Virus Disease 2019 (COVID-19) pandemic, caused by severe acute respiratory syndrome coronavirus 2 (SARS-CoV-2), has lasted more than 3 years worldwide since 11 March 2020. WHO declared the end of COVID-19 as a global health emergency in April 2023, however, COVID-19 is unlikely to end as a global health threat [1]. Vaccines, as the most effective way to improve the health and well-being of populations, have provided the world with great hope in controlling this viral pandemic, leading to a dramatic drop in the risk of infection and hospitalization associated with COVID-19 [2].

Existing COVID-19 vaccine candidates include traditional fully attenuated live and inactivated viruses, protein subunit vaccines, and virus-like particles (VLPs), as well as novel technology nucleic acids, such as mRNA and DNA vaccines [3]. Protein subunit vaccines are the most common approach, accounting for one-third of candidates [2,4]. A recombinant vaccine that comprises the RBD domain of the spike protein has been identified to induce a potent functional antibody response in immunized mice, rabbits, and non-human primates [5]. Effective vaccination depends on the development of strong and durable humoral and cellular immune responses in the vaccinated person [6]. Although universal vaccination has greatly reduced the prevalence and mortality of various diseases, the effectiveness of vaccination has shown great variation at both the individual and group levels [7,8]. There are diverse factors that cause inconsistency in vaccination efficacy, among which environmental exposure of the vaccinated plays a critical role, in addition to the factors regarding vaccines themselves [9]. 

Approximately 20 percent of the world’s population, nearly 1.5 billion people, are infected with helminths. Helminth infections have been a major public health problem, especially for people living in low- and middle-income countries, causing serious health damage and socio-economic losses [10]. As old friends of human coevolution, helminths have a potent ability to overpower our immune system using their offensive (attacking host tissues) and defensive (regulating immune responses) strategies. Helminth infections are associated with broad-spectrum modulation of host immunity to ensure their stable persistence within hosts, thereby also dampening responses to unrelated bystander antigens, such as allergens and immunogens [11]. Helminths have been reported to jeopardize the protective immunity mounted by several vaccines, including BCG [12], hepatitis B virus (HBV) [13,14], influenza [15], human immunodeficiency virus (HIV) [16] and poliovirus vaccine [17]. It is worth noting that deworming drugs can significantly restore the efficacy of some vaccinations [15]. 

The growing lesson of the impact of helminth infections on the effectiveness of various vaccinations raises our concerns about the potential influence of helminth infections on the efficacy of COVID-19 vaccination. It is noteworthy that highly helminth-endemic regions have fewer cases and deaths of COVID-19 [18]. A recent study showed that higher expression of *Ascaris* antibodies is associated with asymptomatic SARS-CoV-2 infections and may reduce the risk of developing severe COVID-19 in Benin, which may be attributed to a decrease in comorbidities and pro-inflammatory markers [19]. It has been reported that helminth co-infection might reduce the severity of COVID-19, which might be related to Th2 and regulatory T cell (Treg) polarization and reduced hyperinflammation caused by helminths [20,21]. On the contrary, another group has found that helminth infection may inhibit an effective immune response to SARS-CoV-2 during the early infection, thereby increasing COVID-19 morbidity and mortality [22]. Therefore, the interaction between helminths and SARS-CoV-2 during coinfection has been inconclusive. Furthermore, helminths have been reported to reduce Th1- and Th17-induced antiviral activity and vaccination efficacy [23]. Considering previous findings on the effects of parasite infections on the efficacy of other respiratory virus vaccines [24,25,26], it can be hypothesized that helminths might impact immune responses to COVID-19 vaccines in endemic populations. In this scenario, assessing the effectiveness of vaccines in people infected with helminths may provide critical information for selecting the optimal strategy for COVID-19 vaccination.

In the current study, a mouse model of trichinosis, a common zoonotic disease worldwide, was used to investigate the effects of different stages of *T. spiralis* infection on the RBD protein vaccine and the related immunological mechanism. Furthermore, we investigated whether deworming with albendazole (ALB) could rescue the inhibitory effect of *T. spiralis* infection on the efficacy of the RBD vaccination in mice.

## 2. Materials and Methods

### 2.1. Animals and Parasites

Female BALB/c mice, 6 to 8 weeks old, were obtained from Liaoning Changsheng Biotechnology Company (Liaoning, China). Kunming mice (female, aged 6 to 8 weeks) were obtained from the Hubei Province Center for Disease Control and Prevention (Wuhan, China). All mice were maintained in a standard specific pathogen-free animal facility. The life cycle of *T. spiralis* was maintained by serial passage in Kunming mice (administered orally with 100 larvae of *T. spiralis* in 0.2 mL of sterilized 0.9% saline) at 6-month intervals. Infected Kunming mice carcasses were collected for determination of skeletal muscle larvae. 

### 2.2. Infection, Vaccination and Treatment

Female BALB/c mice were randomly divided into 8 groups. We grouped mice for the enteric phase experiments as follows: control (Con), immunization (I), *T. spiralis* enteric phase + immunization (E + I), enteric phase + ALB treatment + immunization (A + E + I). We grouped mice for the muscular phase experiments: control (Con), immunization (I), *T. spiralis* muscular phase + immunization (M + I), muscular phase + ALB treatment + immunization (A + M + I). Each mouse from the E + I, A + E + I, M + I, and A + M + I groups was administered by oral gavage with 300 larvae of *T. spiralis* in 0.2 mL of sterilized saline, as previously described [27]. The intestinal phase of *T. spiralis* is reported to occur around day 7 post-infection (pi), and the muscular phase around day 42 pi. Therefore, in order to observe the effects of the two stages on vaccination effectiveness, the first dose of vaccination was selected on the 7th and 42nd day pi, respectively, that is, mice from groups I, E + I, and A + E + I were first immunized on the 7th day pi while mice from groups I, M + I, and A + M + I received the first immunization on the 42nd day pi. The immune agent was 2019-nCoV-RBD antigen (K1516, Nanjing Okai Biotechnology Co., Nanjing, China), diluted to 5 μg/mouse/dose or 10 μg/mouse/dose in 25 μL of phosphate buffered saline (PBS) and mixed with the same volume of alum adjuvant (P77161, Thermo Fisher Scientific, Waltham, USA). Thus, 50 μL of immunogens for each mouse was intramuscularly injected into the thigh muscles of mice 3 times with an interval of 2 weeks [28]. The 1st immunization dose was 10 μg RBD (10 μg RBD in 25 μL PBS plus 25 μL of alum adjuvant) per mouse and 5 μg RBD (5 μg RBD in 25 μL PBS plus 25 μL of alum adjuvant) per mouse for the 2nd and 3rd ones, respectively. Mice from the control (Con) without infection were injected with the same volume of PBS at the corresponding time points. For each infected mouse from the A + E + I and A + M + I groups, ALB (H12020496, SK&F, Tianjin, China) was administrated by oral gavage at a dose of 50 mg/kg body weight for 3 consecutive days starting from day 2 pi [29]. Tail vein blood was taken the day before each immunization. Mice were euthanized at 2 weeks after the final immunization, and blood, bronchoalveolar lavage fluid (BALF), spleen, inguinal lymph nodes, and diaphragm were collected for assessment. 

### 2.3. Enzyme-Linked Immunosorbent Assay (ELISA) of RBD-Specific Antibody Titers

Ninety-six-well plates (BIOFIL) were coated with 1 μg/mL recombinant RBD proteins overnight at 4 °C. After washing with the wash buffer (TBS containing 0.05% (*v*/*v*) polysorbate 20) 3 times, the plates were then blocked with TBS with 1% (*w*/*v*) BSA for 1 h at 37 °C. And plates were incubated with serially diluted mouse serum or BALF for 1 h at 37 °C, following being washed thrice. Next, the plates were incubated with goat anti-mouse IgG (1:25,000 diluted, A21010, Abbkine, Wuhan, China), IgM ((1:10,000 diluted, SA00012-6, Proteintech, Rosemont, IL, USA), sIgA ((1:1000 diluted, SA00012-7, Proteintech), IgG1 (1:10,000 diluted, SA00012-1, Proteintech), and IgG2a (1:10,000 diluted, SA00012-2, Proteintech), respectively. Horseradish peroxidase (HRP)-conjugated secondary antibodies were added and kept for 1 h at 37 °C. Following 5 additional washes, 3,3′,5,5′-tetramethylbenzidine (TMB, Solarbio, Beijing, China) substrate was added and incubated at room temperature in the dark for 10 min. Finally, reactions were stopped by the stop solution (Solarbio). And absorbance at 450 nm was detected using a microplate reader.

### 2.4. ELISA for Cytokines

Concentrations of IFN-γ (Neobioscience), IL-4 (BOSTRE), and IL-10 (BOSTRE) were measured with ELISA kits according to the manufacturer’s instructions.

### 2.5. Detection of Antibody Neutralization Activity

Detection of total neutralizing antibodies to SARS-CoV-2 in mouse serum was performed with a SARS-CoV-2 (2019-nCoV) Neutralization Antibody Screening ELISA Kit (BSKV0004, Bioss, Beijing, China), according to the manufacturer’s instructions. In brief, 50 μL of serum samples, positive (prepared from mouse anti-SARS-CoV-2 neutralization antibody IgG) and negative controls were added to each well in a microplate pre-coating with ACE2 receptor protein, respectively, then mixed with an equal volume of HRP-conjugated recombinant SARS-CoV-2 RBD fragment solution. Subsequently, the plate was incubated for 30 min at room temperature. After 5 times washing, substrate solution was added to each well and incubated for 15 min in the dark. Then the reaction was stopped by the addition of the stop solution and absorbance at 450 nm was evaluated. The percentage of signal inhibition was calculated as follows: Inhibition Rate = (1 − OD value of Sample/OD value of Negative Control) × 100%.

### 2.6. Flow Cytometry

Single-cell suspensions were isolated from the spleen and inguinal lymph nodes. Cells were stained in PBS buffer containing LIVE/DEAD reagent (eBioscience, Waltham, USA) and incubated at room temperature for 25 min in the dark. After washing with PBS buffer, single cell suspensions were Fc blocked with anti-CD16/CD32 monoclonal antibody (BioLegend, San Diego, USA) in FACS buffer for 10 min. Next, cells were stained with antibodies for 30 min on ice. For T lymphocyte staining, antibodies included fluorochrome-conjugated anti-mouse CD4-FITC, CD8-PerCP, anti-mouse Foxp3-PE, and IL-4-PE antibodies (BioLegend). For germinal center (GC) B cell staining, the involved antibodies were PerCP-B220, AF 647-GL7, and FITC-CD95 (BioLegend) [30]. Cells were analyzed by a Sony ID7000 flow cytometer. All data analysis was performed using FlowJo software (Version 9.0). CD4+IL-4+ cells were recognized as Th2 cells, CD4+Foxp3+ cells as Treg cells, and B220^+^GL7^hi^CD95^hi^ as GC B cells.

### 2.7. Statistical Analysis

All results were presented as means ± standard error of the means (SEMs). Statistical analysis was performed using GraphPad Prism 8.0 software. One-way ANOVA tests were performed to assess statistical significance, followed by Tukey’s post-test. Non-parametric multiple comparisons using the Kruskal–Wallis test and Dunn’s post-test were employed in special cases where needed. *p* values less than 0.05 were considered to be statistically significant (* *p* < 0.05, ** *p* < 0.01, *** *p* < 0.001, **** *p* < 0.0001).

## 3. Results

### 3.1. T. spiralis Infection Inhibits the Production of Anti-RBD-Specific Antibodies in Immunized Mice

**Figure 1:**
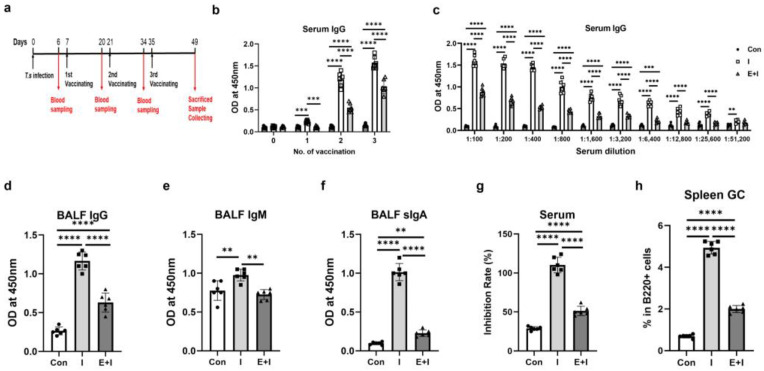
*T. spiralis* infection inhibits the production of anti-RBD-specific antibodies in mice vaccinated at the enteric stage. Each mouse from the enteric phase + immunization group (E + I) was administered orally with 300 larvae of *T. spiralis*. Mice in the immunization (I) and E + I groups were injected intramuscularly with RBD recombinant protein 3 times in the thigh muscle starting from the 7th day after infection, with an interval of 2 weeks between each inoculation. The control (Con) without infection was injected with the same volume of PBS at the corresponding time point. Mice were euthanized at 2 weeks after the 3rd immunization. (**a**) A schematic timeline of the enteric phase experiment protocol; (**b**) Dynamics of anti-RBD-specific IgG in serum (1:100 diluted); (**c**) Titers of serum anti-RBD-specific IgG in vaccinated mice at 2 weeks after the 3rd immunization. Levels of anti-RBD IgG (**d**), IgM (**e**) and sIgA (**f**) in BALF; (**g**) Levels of neutralizing antibody (indicated as inhibition rates) in serum (1:100 diluted); (**h**) Percentages of GC B cells in spleen B cells. Data are expressed as means ± SEMs based on 6 mice in each group. Shown are representative results out of 2 independent experiments. ** *p* < 0.01; *** *p* < 0.001; **** *p* < 0.0001.

**Figure 2:**
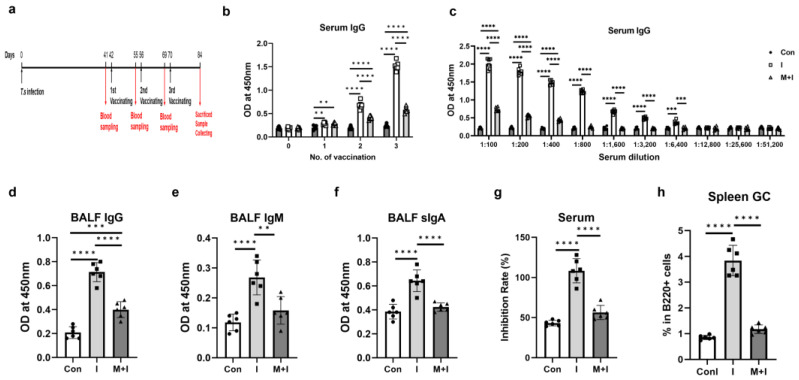
*T. spiralis* infection inhibits the production of anti-RBD-specific antibodies in mice vaccinated at the muscular stage. Each mouse from the muscular phase + immunization group (M + I) was administered orally with 300 larvae of *T. spiralis*. Mice in the immunization (I) and M + I groups were injected intramuscularly with RBD recombinant protein 3 times in the thigh muscle starting from the 42nd day after infection, with an interval of 2 weeks between each inoculation. The control (Con) without infection was injected with the same volume of PBS at the corresponding time point. Mice were euthanized at 2 weeks after the 3rd immunization. (**a**) A schematic timeline of the enteric phase experiment protocol; (**b**) Dynamics of anti-RBD-specific IgG in serum (1:100 diluted); (**c**) Titers of serum anti-RBD-specific IgG in vaccinated mice at 2 weeks after the 3rd immunization. Levels of anti-RBD IgG (**d**), IgM (**e**) and sIgA (**f**) in BALF; (**g**) Levels of neutralizing antibody (indicated as inhibition rates) in serum (1:100 diluted); (**h**) Percentages of GC B cells in spleen B cells. Data are expressed as means ± SEMs based on 6 mice in each group. Shown are representative results out of 2 independent experiments. ** *p* < 0.01; *** *p* < 0.001; **** *p* < 0.0001.

A mouse model infected with *T. spiralis* was established and used to examine the effects of two stages of trichinosis, the enteric stage and the muscular stage, on the efficiency of COVID-19 vaccination, respectively (Figure 1a and Figure 2a). Serum levels of RBD-specific antibodies in mice were detected at different time points to explore the dynamic changes in antibody levels. 

For the enteric stage, as Figure 1b depicts, serum RBD-specific IgG levels rose after the 1st immunization and peaked 2 weeks after the 3rd immunization. The enteric phase + immunization group had much lower levels of IgG than the immunization at all time points after vaccination (Figure 1b), and the IgG level of the enteric phase + immunization group was still lower than that of the immunization group even when the serum was diluted to 1:25,600 at 2 weeks after the 3rd immunization (Figure 1c). Given that the respiratory mucosal immune response is the first line of defense against SARS-CoV-2 infection, next, the levels of antibodies in BALF were detected. The immunization group had higher levels of RDB-specific IgG, IgM, and secreted IgA (sIgA) in BALF compared to the control group, while the enteric phase + immunization group had lower levels of these three antibodies related to the immunization (Figure 1d–f). Generating potent neutralizing antibodies is a unifying goal of vaccination since the best effect of vaccination is to induce the production of powerful neutralizing antibodies in hosts [31]; then, we investigated the levels of neutralizing antibodies. The results showed that the enteric phase + immunization group had a lower serum inhibition rate than the immunization group (Figure 1g). GC B cells are the source of effective antibodies required for protective immunity after vaccination [32] and recombinant RBD protein elicits strong GC responses in spleen and lymph nodes in the vaccinated mice [33]. As Figure 1h depicted, the enteric phase significantly reduced the increased GC B cells elicited by RBD vaccination in infected mice. 

For the muscular stage, as indicated in Figure 2b, serum RBD-specific IgG levels continued to rise after the 1st immunization and reached a peak by the end of the experiment. The IgG levels in the muscular phase + immunization group were significantly lower than those in the immunization group after the 2nd and 3rd immunizations. The IgG level of the muscular phase + immunization group was still lower than that of the immunization group even when the serum was diluted to 1:6400 at 2 weeks after the 3rd immunization (Figure 2c). In addition, the muscular phase + immunization group had much lower BALF levels of IgG, IgM, and sIgA than the immunization (Figure 2d–f). As revealed in Figure 2g, the muscular phase + immunization group had a lower serum level of neutralizing antibodies than the immunization group. Similar to the results in the enteric phase, the percentage of GC B cells in infected mice vaccinated in the muscular phase was significantly lower than that in the vaccination group alone (Figure 2h).

Altogether, these results suggest that both the enteric and muscular stages significantly inhibit the production of RBD-specific antibodies, thereby reducing the efficacy of RBD vaccination in mice, which might be related to the impaired germinal response caused by *T. spiralis* infection, in light of the importance of GC cells for the generation of high-quality antibodies.

### 3.2. Pre-Exposure to the Enteric Phase of Trichinosis Promotes Th2 and Treg Immune Responses in Immunized Mice

**Figure 3:**
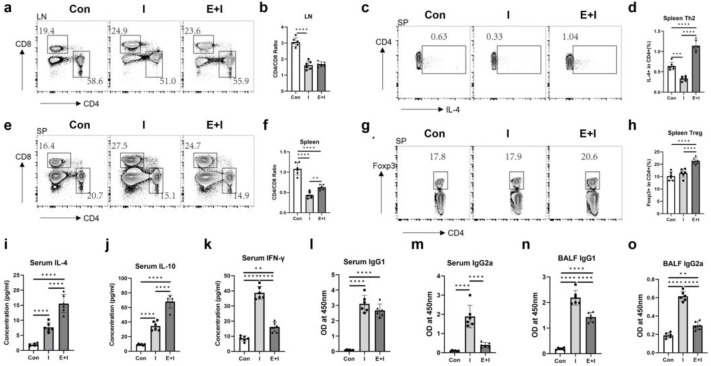
Pre-exposure to the enteric stage of trichinosis promotes Th2 and Treg immune responses in immunized mice. Mice were euthanized at 2 weeks after the 3rd immunization. The spleen and inguinal lymph node were collected and lymphocytes were prepared before staining for flow cytometry. (**a**,**b**) Ratios of CD4+/CD8+ cells in inguinal lymph nodes; (**c**,**d**) Proportions of CD4+IL-4+ T cells in splenic T cells; (**e**,**f**) Ratios of CD4+/CD8+ cells in the spleen; (**g**,**h**) Proportions of CD4+Foxp3+ T cells in splenic CD4+ cells; Concentrations of IL-4 (**i**), IL-10 (**j**), and IFN-γ (**k**) in serum; Serum levels of anti-RBD IgG1 (**l**) and IgG2a (**m**); BALF levels of anti-RBD IgG1 (**n**) and IgG2a (**o**). Data are expressed as means ± SEMs based on 6 mice in each group. Shown are representative results out of 2 independent experiments. Con: the control group; I: the immunization group; E + I: the enteric phase + immunization group. ** *p* < 0.01; *** *p* < 0.001; **** *p* < 0.0001.

Previous studies have highlighted the significant and multifaceted roles of T cells in protective immunity against COVID-19 [34]. Vaccinated subjects have a significantly increased expression of CD8+ T cells, which might reduce the CD4+/CD8+ ratio [35]. As Figure 3a,b,e,f revealed, compared with the control group, both the immunization and the enteric stage + immunization groups had decreased CD4/CD8 ratios in inguinal lymph node and spleen. The enteric stage + immunization had a higher splenic CD4/CD8 ratio than the immunization one (Figure 3e,f). 

Given that helminth antigens can drive modified immune responses characterized by Th2 and regulatory responses [36], we investigated the frequencies and functional cytokines of splenic Th2 and Treg cells. The results indicated that the enteric phase + immunization group had higher proportions of splenic Th2 (Figure 3c,d) and Treg (Figure 3g,h) cells than the immunization group, while the immunization inhibited the splenic Th2 frequency related to the control group (Figure 3c,d). Both the immunization and enteric phase + immunization groups had higher concentrations of serum IL-4 (Figure 3i) and IL-10 (Figure 3j) than the control, and the infection increased IL-4 and IL-10 levels related to the immunization group (Figure 3i,j). In addition, the infection suppressed the increased levels of serum IFN-γ in the mice induced by vaccination (Figure 3k). Analysis of the distinct IgG isotypes showed there were augments of IgG1 and IgG2a titers in both serum and BALF induced by the immunization, whereas the enteric stage harmed IgG1 levels in BALF and IgG2a levels in serum and BALF (Figure 3l–o). Taken together, all the data suggest that pre-exposure to the enteric phase of trichinosis promotes type 2 and Treg immune responses in the immunized mice.

### 3.3. Pre-Exposure to the Muscular Phase of Trichinosis Promotes Th2 Immune Responses in Immunized Mice

**Figure 4:**
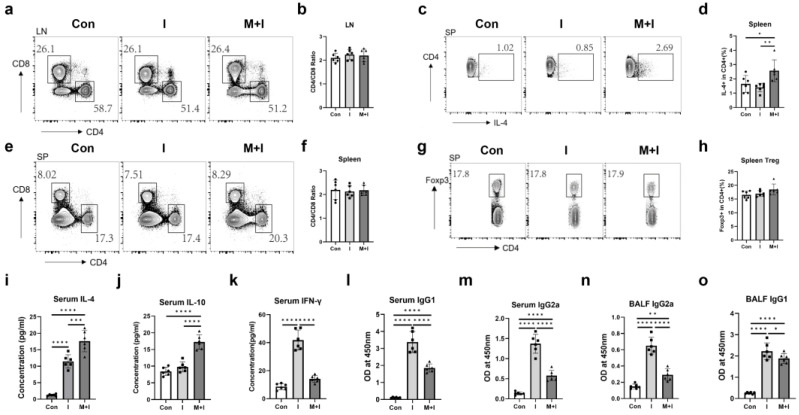
Pre-exposure to the muscular stage of trichinosis promotes Th2 immune responses in immunized mice. Mice were euthanized at 2 weeks after the 3rd immunization. The spleen and inguinal lymph node were collected and lymphocytes were prepared before staining for flow cytometry. (**a**,**b**) Ratios of CD4+/CD8+ cells in inguinal lymph nodes; (**c**,**d**) Proportions of CD4+IL-4+ T cells in splenic T cells; (**e**,**f**) Ratios of CD4+/CD8+ cells in the spleen; (**g**,**h**) Proportions of CD4+Foxp3+ T cells in splenic CD4+ cells; Concentrations of IL-4 (**i**), IL-10 (**j**), and IFN-γ (**k**) in serum; Serum levels of anti-RBD IgG1 (**l**) and IgG2a (**m**); BALF levels of anti-RBD IgG1 (**n**) and IgG2a (**o**). Data are expressed as means ± SEMs based on 6 mice in each group. Shown are representative results out of 2 independent experiments. Con: the control group; I: the immunization group; M + I: the muscular phase + immunization group. * *p* < 0.05; ** *p* < 0.01; *** *p* < 0.001; **** *p* < 0.0001.

Next, we examined the immunological characteristics of the immunized mice pre-exposure to the muscular stage of trichinosis. As indicated in Figure 4a,b,e,f, no differences in CD4/CD8 ratios from murine spleen and inguinal lymph nodes were observed among the three experimental groups. The muscular phase + immunization group had a higher proportion of splenic Th2 cells (Figure 4c,d) than the immunization group, while no difference in Treg (Figure 4g,h) was found between the two groups. Both the immunization and muscular phase + immunization groups had higher concentrations of serum IL-4 (Figure 4i) than the control, and the infection increased IL-4 and IL-10 levels related to the immunization group (Figure 4i,j). In addition, the infection suppressed the increased levels of serum IFN-γ in the mice induced by vaccination (Figure 4k). Analysis of the distinct IgG isotypes showed there were augments in serum and BALF IgG1 and IgG2a titers induced by the immunization, whereas the muscular stage hurt the above antibody levels in various degrees (Figure 4l–o). Collectively, pre-exposure to the muscular phase of trichinosis promotes Th2 immune responses in immunization mice, not affecting Treg immune responses.

### 3.4. ALB Treatment Partially Rescues the Inhibition of T. spiralis Infection on the Production of Anti-RBD-Specific Antibodies in Immunized Mice

**Figure 5:**
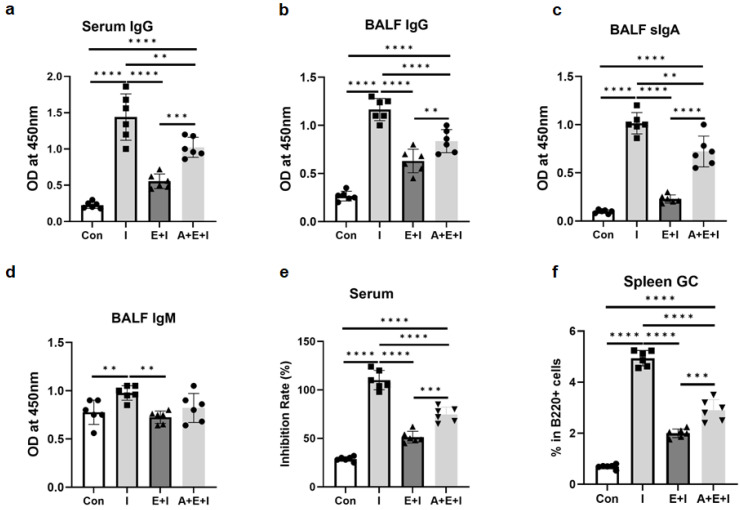
Albendazole (ALB) treatment partially rescues the inhibition of the enteric stage of trichinosis on anti-RBD-specific antibodies in immunized mice. In the enteric stage + ALB treatment + immunization group (A + E + I), ALB was taken orally at a dose of 50 mg/kg body weight for 3 consecutive days starting from day 2 post-infection. Mice were euthanized at 2 weeks after the 3rd immunization. (**a**) Levels of anti-RBD IgG antibodies in serum; Levels of anti-RBD IgG (**b**), sIgA (**c**), and IgM (**d**) in BALF; (**e**) Levels of neutralizing antibody (indicated as inhibition rates) in serum (diluted 1:100); (**f**) Percentages of GC B cells in spleen B cells. Data are expressed as means ± SEMs based on 6 mice in each group. Shown are representative results out of 2 independent experiments. Con: the control group; I: the immunization group; E + I: the enteric phase + immunization group; A + E + I: the enteric phase + ALB treatment + immunization. ** *p* < 0.01; *** *p* < 0.001; **** *p* < 0.0001.

**Figure 6:**
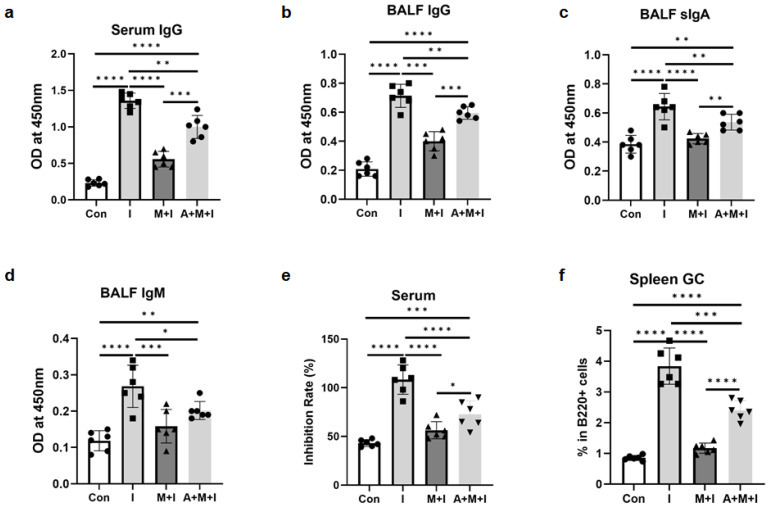
Albendazole (ALB) treatment partially rescues the inhibition of the muscular stage of trichinosis on anti-RBD-specific antibodies in immunized mice. In the muscular stage + ALB treatment + immunization group (A + M + I), ALB was taken orally at a dose of 50 mg/kg body weight for 3 consecutive days starting from day 2 post-infection. Mice were euthanized at 2 weeks after the 3rd immunization. (**a**) Levels of anti-RBD IgG antibodies in serum; Levels of anti-RBD IgG (**b**), sIgA (**c**), and IgM (**d**) in BALF; (**e**) Levels of neutralizing antibody (indicated as inhibition rates) in serum (diluted 1:100); (**f**) Percentages of GC B cells in spleen B cells. Data are expressed as means ± SEMs based on 6 mice in each group. Shown are representative results out of 2 independent experiments. Con: the control group; I: the immunization group; M + I: the muscular phase + immunization group; A + M + I: the muscular phase + ALB treatment + immunization. * *p* < 0.05; ** *p* < 0.01; *** *p* < 0.001; **** *p* < 0.0001.

Since all findings confirmed that *T. spiralis* infection inhibited the efficacy of RBD immunization in vivo, we next attempted to investigate whether ALB deworming before vaccination could rescue the inhibition effect of the infection on vaccine effectiveness. We found that ALB treatment enhanced RBD-specific IgG titers in serum (Figure 5a and Figure 6a) and BALF (Figure 5b and Figure 6b), as well as levels of sIgA (Figure 5c and Figure 6c) and IgM (Figure 5d and Figure 6d) in BALF, in both the enteric stage + immunization and the muscular stage + immunization groups. Results of the neutralizing antibody experiment showed that ALB treatment partially restored the serum inhibition rates in both the enteric stage + immunization and the muscular stage + immunization groups, although antibodies did not reach the levels in the immunization groups (Figure 5e and Figure 6e). Furthermore, the negative impacts of GC B cells elicited by the two stages of infection from vaccinated mice were partially rescued by the ALB treatment (Figure 5f and Figure 6f). Combined, ALB deworming before vaccination could partially rescue the inhibition of *T. spiralis* infection on the production of RBD-specific antibodies in the immunized mice, which might be associated with the positive effect of ALB treatment on the inhibited GC immunity caused by *T. spiralis* infection in mice.

## 4. Discussion

In the current study, using mice infected with *T. spiralis* as an infection model, we studied the effect of helminth infection on the efficacy of the RBD vaccination. Impaired responses were observed when vaccinations were performed in both the enteric and muscular stages of murine trichinosis. Mechanistically, the suppression was associated with a systemic expansion of Treg cells and polarization of Th2 immune responses, as well as inhibited GC immunity. Fortunately, ALB treatment partially restored the inhibition of *T. spiralis* infection on the COVID-19 vaccination. 

It has been confirmed that RBD vaccination can effectively produce high levels of protective antibodies against SARS-CoV-2 in BALB/c and C57BL/6 mice and primates [37,38]. In line with these findings, our study showed that intramuscular injection of RBD protein could induce high serum levels of anti-RBD-specific antibodies and neutralizing antibodies in BALB/c mice, suggesting an effective humoral immune response after vaccination.

A growing body of studies, both in animal models and human studies, has shown that helminth infections profoundly impact immune responses to vaccination in hosts, although different helminth species vary in the effects and mechanisms of different vaccines [39]. Prior *Nippostrongylus brasiliensis* infection induces a compromised immune response to *Salmonella typhimurium* vaccine, evidenced by decreased antibody titer in mice [40]. The immune responses to bystander Ag of mycobacteria and plasmodia are significantly inhibited in Indonesian children infected with geohelminths [41]. Another group reports that chronic *Schistosoma mansoni* infection impairs the persistence of vaccine-specific antibody responses in poliovirus-vaccinated humans and mice [17]. Our previous findings suggest that the enteric stage of *T. spiralis* infection results in a reduced immune response to hepatitis B surface antigen (HBsAg); in contrast, the muscular stage does not affect the effectiveness of hepatitis B virus (HBV) vaccination [13]. Here, we found that both the enteric and muscular phases of trichinosis resulted in decreased levels of IgG (the most abundant protective antibody) in serum and BALF, along with reduced serum neutralizing antibodies after vaccination. This observation is partially in line with our publication of *T. spiralis* infection being associated with reduced responses to HBV vaccine in mice [13]. Muscular larvae and adults generally induce Th2 polarization in the enteric phase, while new-borne larvae are considered to induce a mixed Th1/Th2 immune response in the muscular phase [42]. It is well known that vaccine efficiencies are dependent on the immune state of the individual. The modified immune responses induced by antigens of different stages of *T. spiralis*, play a crucial role in determining the outcome of vaccination. This may be the reason why the enteric phase and muscular phase of *T. spiralis* have different effects and underlying mechanisms on the RBD vaccination effect. Moreover, different effects of muscular stage on HBV vaccination (no impact) and RBD vaccination (inhibited impact) might be related to types of vaccines and immune responses induced by various vaccines. In addition, the pre-existing trichinosis impaired the level of anti-RBD sIgA antibody in BALF, indicating a damaged mucosal immunity [43]. Furthermore, both stages of *T. spiralis* infection led to decreased proportions of splenic GC cells related to the immunization groups, indicating impaired humoral responses in mice. It has been shown that potent GC responses are strongly correlated with neutralizing antibody production [44]. Therefore, prior *T. spiralis* infection not only compromised systemic immune responses but also suppressed local mucosal immunity in the RBD-vaccinated mice.

Vaccine-induced IgG subclasses have been reported to reflect the profile of protective immunity in the vaccinated mouse model [37]. Investigating how helminths affect antibody induction can be informative in identifying how best to employ vital life-saving vaccines [40]. Therefore, we further enumerated serum subtypes of IgG antibodies in serum and BALF. The results showed that compared with the immunization group, both the enteric and muscular stages resulted in decreased levels of RBD-specific IgG2a (Th1-reflecting isotype), along with inhibited levels of IgG1 (Th2-reflecting isotypes) in serum and BALF. Therefore, pre-exposure to *T. spiralis* infection suppressed both type 1 and 2 antibodies in the immunized mice.

An efficient Th1 response is required to prevent most viral diseases successfully [37,45]. However, helminth infections are generally characterized by a Th2 shift type response as well as a regulatory response. The efficiencies of vaccines are dependent on the immune state of the immunized and the Th2-dominant immune response attenuates vaccine responses [46]. In the present study, we demonstrated that both the enteric and muscular stages of *T. spiralis* infection led to Th2 polarization immunity, as well as an enhanced Treg response in the enteric stage. The global population harbors helminth parasites that can impair human immune responses to several other vaccines, which occurs through the involvement of interleukin (IL)-10 and regulatory T cells [47]. It has been indicated that this sustained suppressive state against influenza vaccination is correlated to Treg expansion in *Litomosoides sigmodontis*-infected mice [15]. Another group reports that concurrent infection with *L. sigmodontis* reduces the quantity and quality of antibody responses to vaccination against seasonal influenza in BALB/c and C57BL/6 mice, which is related to a systemic and sustained expansion of IL-10-producing Treg cells [26]. An in vitro study showed that Th cell-mediated immune responses to SARS-CoV-2 are mitigated in the presence of helminth antigens [48]. The mechanisms of helminth attenuation of vaccine responses are not fully known, but a role for Treg, IL-10, or follicular helper T cells has been proposed [25]. Given the interaction between helminths and SARS-CoV-2 during coinfection is inconclusive or even conflicting [18,19,20,21,22], the regulation of helminths on the viral immunity is complicated. Previous studies indicate that the reduction in COVID-19 disease severity may be related to Th2 and Treg polarization and reduced hyperinflammation caused by helminths during the co-infection [20,21], which is in line with our results that *T. spiralis* infection inhibits the efficacy of the COVID-19 vaccine, associated with its polarization of Th2 and Treg responses. Certainly, further experimental evaluations are required to decisively elucidate the underlying mechanism. The screening and identification of effective molecules from *T. spiralis* soluble antigen are ongoing in our lab, and we will further explore how these active components regulate T cell immunity and thus inhibit the vaccination effectiveness.

Various studies have attempted to rescue vaccination responses in helminth-endemic areas through deworming, with inconsistent results. Some observe a significant improvement in vaccination responses after deworming treatment [15,49,50,51,52,53], while others report partial restoration [54] or no obvious effect of deworming [55,56]. In this study, ALB treatment could partially reverse the inhibition effect of *T. spiralis* infection on the efficacy of the RBD vaccine. It has been found that two doses of ALB administration before vaccination with an attenuated oral cholera vaccine are sufficient to double seroconversion rates [57], while a single dose of 400 mg of ALB before influenza immunization has no significant effect on influenza vaccine immunogenicity in children from Gabon [52]. A recent study indicates that praziquantel treatment mitigates the parasite’s negative influences on poliovirus vaccine immunity in infected school-aged children and mice with *S. mansoni* [17]. We also found that parasite clearance following ALB treatment partially restored decreased GC cells caused by *T. spiralis* infection in the vaccinated mice. These observations suggest that the negative impact of *T. spiralis* on RBD vaccination effectiveness might be rooted in its inhibition of GC immune response in mice. Therefore, before implementing the COVID-19 vaccines, mass deworming could be a pharmacological intervention to improve anti-SARS-Cov-2 protection in helminth-infected individuals, particularly in tropical countries, obviously requiring in-depth investigation and confirmation.

There were two limitations in the current study. Firstly, an ALB-only group in these treatment experiments was lacking. The Hu group found that ALB may have an anti-tumor effect in combination therapy with the immune checkpoint inhibitor or oncolytic virus M1 through activating CD8+ T cells [58]. Another group reported that ALB therapy provokes secretions of IFN-γ and IL-6 in infected swine with *Taenia solium*, which is highly related to the dead cysticerci [59]. However, ALB at doses of 2.5 and 5.0 mg/kg does not affect the immune response of murine cellular and humoral type [60]. Therefore, immunomodulatory effects of ALB and its underlying mechanism are closely related to the disease model and the dose used. It is necessary to explore whether ALB has immunomodulatory effects in mice at a dose of 50 mg/kg. Secondly, the persistence of antibody responses or the effect of duration on vaccine efficacy was not detected. The results of a recent meta-analysis regarding parasite infections and immunization reveals that chronic helminthiasis has a more negative impact on immunization than acute disease [57]. Importantly, the effects of helminths are prolonged, with attenuated vaccine responses of up to 16 weeks after infection clearance in mice [25]. These findings warrant further investigation of the long-term effects of helminth infection on vaccination efficiency. 

## 5. Conclusions

To the best of our knowledge, this is the first experimental report about the impact of helminth infection on the RBD vaccination, highlighting the risk of failed vaccinations due to helminth infection. In summary, *T. spiralis* infection inhibits the efficacy of the RBD vaccine, which is associated with its polarization of Th2 and Treg responses. ALB treatment can partially reverse the inhibitory effect of *T. spiralis* infection on the efficiency of RBD vaccination, which is related to the enhanced GC B cells by ALB administration. Given the widespread prevalence of helminth infections around the world, integrating our findings into the global antiviral vaccination strategy, deworming therapy needs to be considered when implementing COVID-19 vaccination strategies.

## Data Availability

All data generated or analyzed during the current study are included in the article.
